# Supplementary figures and images for: Female philopatry may influence antipredatory behavior in a solitary mammal
Source: PeerJ. 2025 Mar 20;13:e18933. doi: 10.7717/peerj.18933 (PMC11930214; doi:10.7717/peerj.18933)

$d = 10$

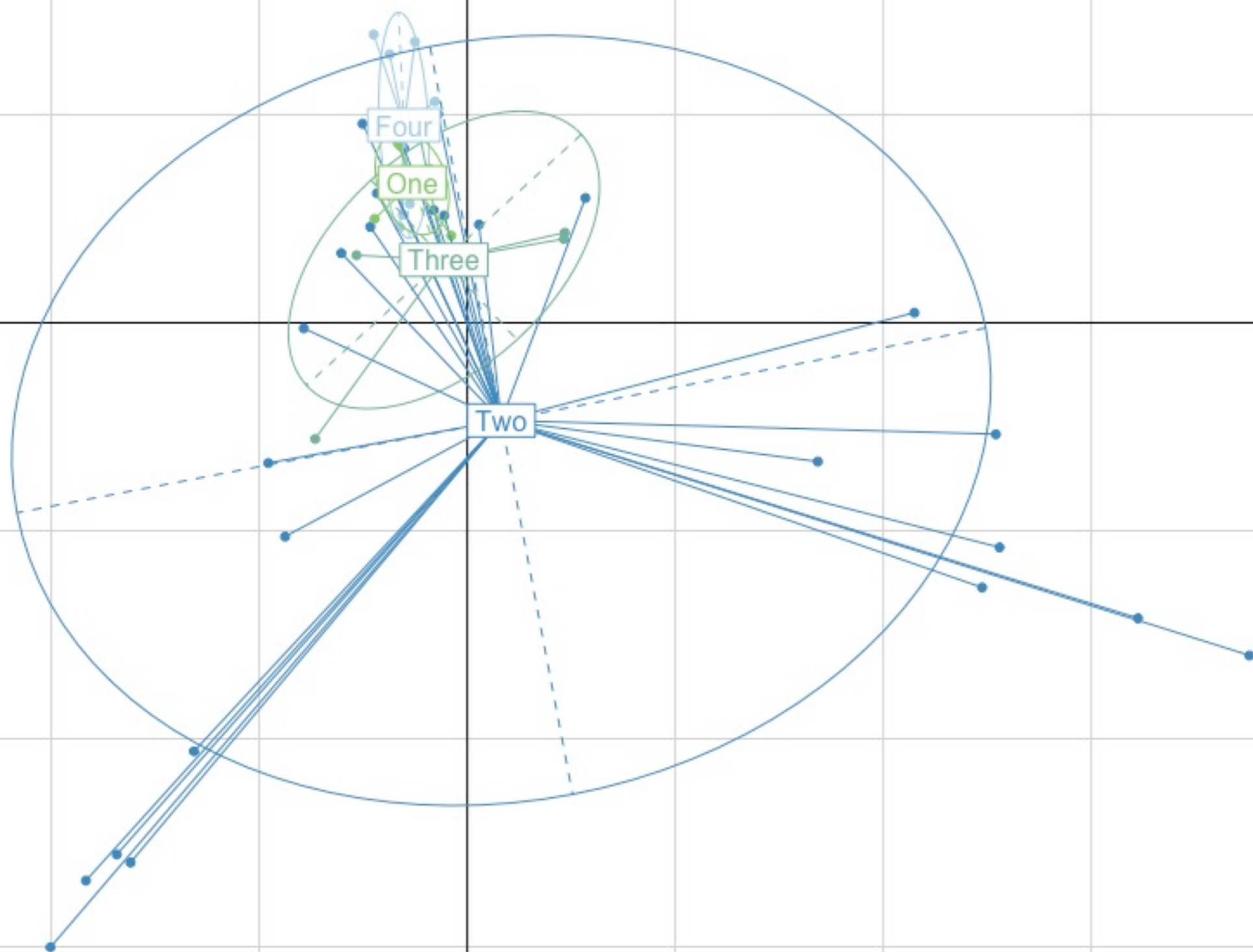

Supplement: Supplemental Information 1 — Principal Component Analysis showing that all four trapping locations likely sampled animals within a single population. [file peerj-13-18933-s001.pdf]

**Value of BIC  
versus number of clusters**

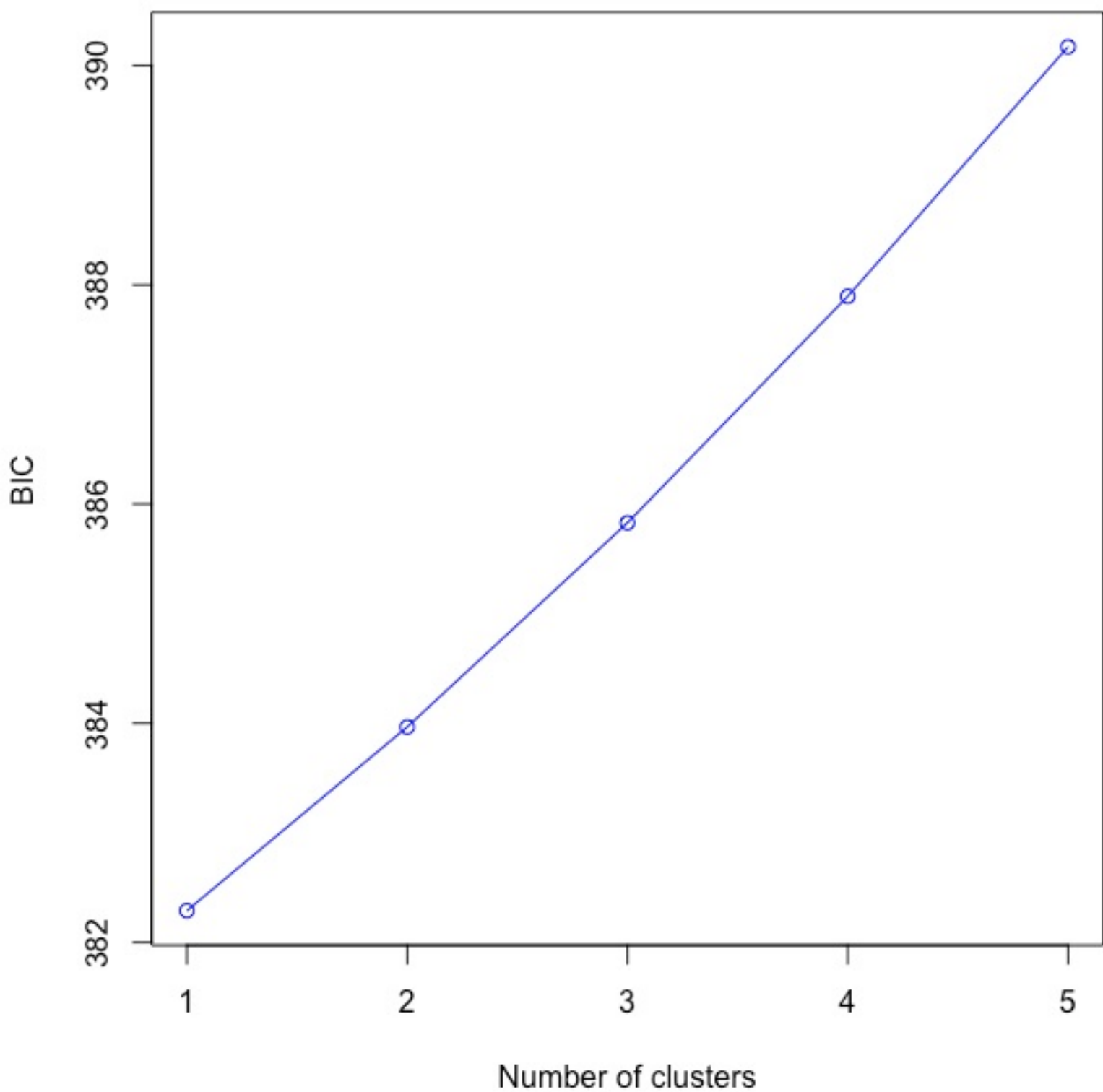

Supplement: Supplemental Information 2 — The number of clusters indicated by the lowest BIC suggests that all individuals can be grouped genetically into a single cluster. [file peerj-13-18933-s002.pdf]

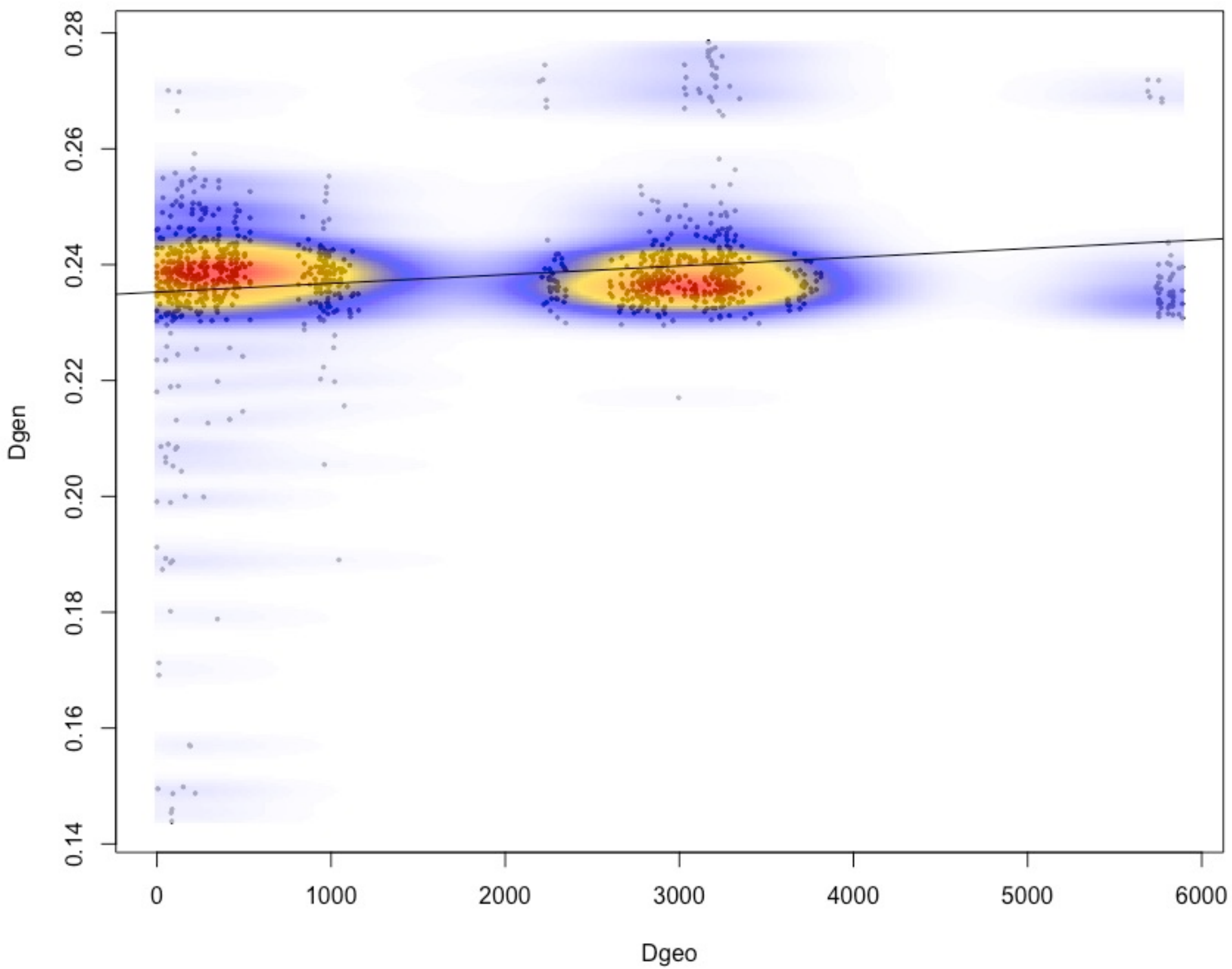

Supplement: Supplemental Information 3 — Scatterplot showing the relationship between geographic distance (spatial Euclidean in meters) and genetic distance (inverse proportion of alleles shared between individuals) of all sampled Harris’s antelope squirrels (n = 47). Colors represent the relative density of points: red showing higher density, yellow medium density, and blue lower density. A Mantel test showed a significant relationship between geographic and genetic distance (R = 0.18, p < 0.001). [file peerj-13-18933-s003.pdf]

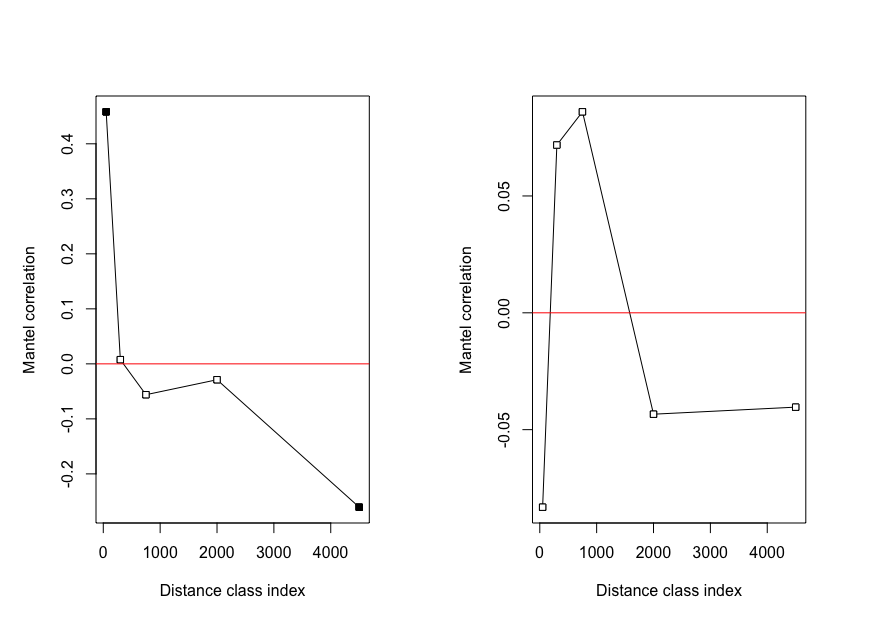

Supplement: Supplemental Information 4 — Mantel correlogram showing how the correlation between geographic and genetic distance change with distance class. Female antelope squirrels exhibited a positive correlation between geographic and genetic distance at close distances and a negative correlation at larger distances. Males showed very low correlations between geographic and genetic distance across distance classes. Black points represent significant (p < 0.05) Mantel correlations; white points represent nonsignificant Mantel correlations. Note the difference in scale on the y-axis. [file peerj-13-18933-s004.png]
