# Supplementary material for: Female philopatry may influence antipredatory behavior in a solitary mammal
Source: PeerJ. 2025 Mar 20;13:e18933. doi: 10.7717/peerj.18933 (PMC11930214; doi:10.7717/peerj.18933)
Supplement: Supplemental Information 5 — Summary of Mantel correlogram analysis showing how the relationship between female-female genetic distance and geographic distance changes with distance class. Bold values indicate significance. Class index represents the median of each distance class. [file peerj-13-18933-s005.docx]

Table S1: **Results of Mantel correlogram analysis for female Harris’s antelope squirrels.** Summary of Mantel correlogram analysis showing how the relationship between female-female genetic distance and geographic distance changes with distance class. Bold values indicate significance. Class index represents the median of each distance class.

| Distance class | Class index | n | Mantel correlation | p - value (Mantel) | p - value (corrected) |
| --- | --- | --- | --- | --- | --- |
| D.cl.1 | 50 | 86 | 0.46 | 0.001 | **0.001** |
| D.cl.2 | 300 | 268 | 0.01 | 0.440 | 0.440 |
| D.cl.3 | 750 | 58 | -0.06 | 0.175 | 0.350 |
| D.cl.4 | 2000 | 162 | -0.03 | 0.315 | 0.630 |
| D.cl.5 | 4500 | 238 | -0.26 | 0.001 | **0.005** |
