# Supplementary material for: Female philopatry may influence antipredatory behavior in a solitary mammal
Source: PeerJ. 2025 Mar 20;13:e18933. doi: 10.7717/peerj.18933 (PMC11930214; doi:10.7717/peerj.18933)
Supplement: Supplemental Information 6 — Summary of Mantel correlogram analysis showing how the relationship between male-male genetic distance and geographic distance changes with distance class. Class index represents the median of each distance class. [file peerj-13-18933-s006.docx]

Table S2: **Results of Mantel correlogram analysis for male Harris’s antelope squirrels.** Summary of Mantel correlogram analysis showing how the relationship between male-male genetic distance and geographic distance changes with distance class. Class index represents the median of each distance class.

| Distance class | Class index | n | Mantel correlation | p - value (Mantel) | p - value (corrected) |
| --- | --- | --- | --- | --- | --- |
| D.cl.1 | 50 | 24 | -0.08 | 0.103 | 0.103 |
| D.cl.2 | 300 | 64 | 0.07 | 0.239 | 0.239 |
| D.cl.3 | 750 | 46 | 0.09 | 0.203 | 0.406 |
| D.cl.4 | 2000 | 80 | -0.04 | 0.321 | 0.609 |
| D.cl.5 | 4500 | 92 | -0.04 | 0.334 | 0.812 |
